# Prevalence of anti-*Trypanosoma cruzi* and anti-*Leishmania infantum* antibodies in domestic dogs from Tremedal, Bahia: insights from the Oxente Chagas Bahia Project

**DOI:** 10.1186/s13071-025-06744-9

**Published:** 2025-03-21

**Authors:** Tycha Bianca Sabaini Pavan, Larissa Carvalho Medrado Vasconcelos, Isabela Machado Serrano, Denis Augusto Argolo Campos, Ângelo Antônio Oliveira Silva, Randrin Queiroz Viana Ferreira, Daniel Dias Sampaio, Isadora Cristina de Siqueira, Fred Luciano Neves Santos

**Affiliations:** 1 Advanced Public Health Laboratory, Gonçalo Moniz Institute, Oswaldo Cruz Foundation, Salvador, Bahia Brazil; 2 Interdisciplinary Research Group in Biotechnology and Epidemiology of Infectious Diseases (GRUPIBE), Gonçalo Moniz Institute, Oswaldo Cruz Foundation, Salvador, Bahia Brazil; 3 Chagas Disease Translational Research Program (Fio-Chagas), Oswaldo Cruz Foundation, Rio de Janeiro, Rio de Janeiro Brazil; 4 Laboratory of Investigation in Global Health and Neglected Diseases, Gonçalo Moniz Institute, Oswaldo Cruz Foundation, Salvador, Bahia Brazil

**Keywords:** Chagas disease, Visceral leishmaniasis, *Trypanosoma cruzi*, *Leishmania infantum*, Dogs, One Health, Zoonosis

## Abstract

**Background:**

Chagas disease (CD) and visceral leishmaniasis (VL) are two important zoonotic diseases that present significant public health challenges in Latin America. Domestic dogs, due to their close contact with humans, serve as key reservoirs for both *Trypanosoma cruzi* (the causative agent of CD) and *Leishmania infantum* (the causative agent of VL), making them important sentinels in disease surveillance. This study, conducted as part of the Oxente Chagas Bahia Project, aimed to assess the seroprevalence of anti-*T. cruzi* and anti-*L. infantum* antibodies in domestic dogs from Tremedal, Bahia, Brazil.

**Methods:**

Serum samples from 17 dogs were analyzed using indirect enzyme-linked immunosorbent assay (ELISA) (using recombinant antigens (IBMP-8.1, IBMP-8.2, IBMP-8.3, IBMP-8.4) for *T. cruzi* and the TR DPP® rapid test and ELISA for *L. infantum*.

**Results:**

The results showed that 5.9% (1/17) of the dogs tested were seropositive for *T. cruzi*, indicating the presence of the parasite in the region. Similarly, 5.9% (1/17) of the dogs were confirmed to be positive for *L. infantum* by ELISA, although the results of the TR DPP® test initially suggested a higher prevalence (41.2%), highlighting the risk of false-positive results.

**Conclusions:**

These findings underscore the critical role of dogs in CD and VL surveillance, given their involvement in both domestic and peridomestic transmission cycles. The study also emphasizes the need for confirmatory testing to ensure diagnostic accuracy, which will contribute to more effective disease control strategies in endemic areas. This work highlights the importance of a One Health approach in which human and animal health are closely monitored to mitigate the transmission of zoonotic diseases.

**Graphical Abstract:**

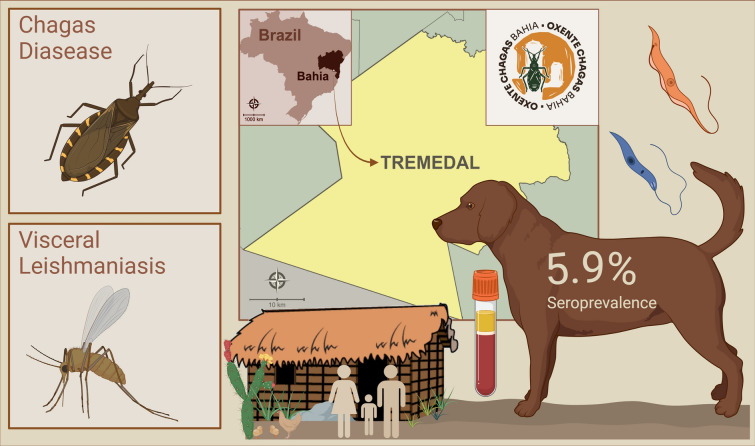

## Background

Chagas disease (CD) and visceral leishmaniasis (VL) are life-threatening neglected zoonotic diseases caused by the hemoflagellate protozoans *Trypanosoma cruzi* [1] and *Leishmania infantum*, respectively. Management of these diseases poses substantial public health challenges. CD affects an estimated 6–7 million people across 21 Latin American countries, resulting in approximately 12,000 deaths annually [2] while VL affects between 200,000 and 400,000 people globally, with 90% of new cases concentrated in 10 countries, including Brazil [3].

In endemic areas, the primary transmission route of *T. cruzi* is though contact with the excreta of infected blood-sucking triatomine bugs (commonly known as kissing bugs), which enter the host via mucosal surfaces, skin abrasions or insect bites. Secondary transmission routes, including vertical transmission, blood transfusions, organ transplantation and ingestion of contaminated food, can also play significant roles depending on the endemicity of the parasite and local public health policies [4]. For *L. infantum*, transmission primarily occurs through the bite of infected phlebotomine sand flies, although non-vectorial transmission routes, such as sexual, vertical and blood transfusion, have been documented [5, 6].

Various domestic and wild mammals serve as reservoirs for *T. cruzi* and *L. infantum*. Among these, domestic dogs are particularly important due to their close association with humans in household environments. Dogs play a crucial role in the domestic and peridomestic transmission cycles of these pathogens, acting as significant reservoirs [7–9]. Infected dogs are recognized as natural sentinels that signal the presence of *T. cruzi* or *L. infantum* in specific areas [8–11], which in turn elevates the risk of human infection [12, 13]. This cycle underscores the importance of vigilant monitoring and the control of infected animals to limit the spread of CD and VL.

In this context, we investigated the prevalence of anti-*T. cruzi* and anti-*Leishmania* spp. antibodies in domestic dogs from Tremedal, an endemic area in the Brazilian state of Bahia. The aim of this study was to deepen our understanding of the role dogs play in the transmission of these pathogens and to inform more effective disease control strategies. This study is part of the Oxente Chagas Bahia Project, an initiative designed to validate a rapid diagnostic test for humans in a real-world setting, providing crucial data on the test’s diagnostic performance and cost-effectiveness, while addressing CD and VL within a One Health framework [14].

## Methods

### Study area

This study was conducted in Tremedal, a municipality in the state of Bahia, Northeastern Brazil (14°58′33″S, 41°24′39″W), as part of the Oxente Chagas Bahia Project [14]. Tremedal is primarily a rural area where agriculture serves as the main economic activity. The region experiences a semi-arid climate and is characterized by typical backlands vegetation. Covering an area of approximately 1679.6 km^2^, Tremedal has an estimated population of 16,296 inhabitants, with a population density of 8.11 inhabitants/km^2^ [15]. Economically, Tremedal ranks 2089th among Brazil's 5570 municipalities and 211th among Bahia's 417 municipalities in terms of per capita income [15]. Despite efforts to eradicate *Triatoma infestans*, the primary vector of *T. cruzi*, residual foci of this vector were last observed in Tremedal in 2011 [16]. In recent years, other triatomine species, such as *Triatoma sordida*, *Panstrongylus geniculatus*, *Triatoma pseudomaculata*, *Panstrongylus lutzi*, *Panstrongylus geniculatus* and *Panstrongylus diasi*, have been captured in the region, sustaining conditions for the continued transmission of *T. cruzi* [17].

### Study population

**Fig. 1:**
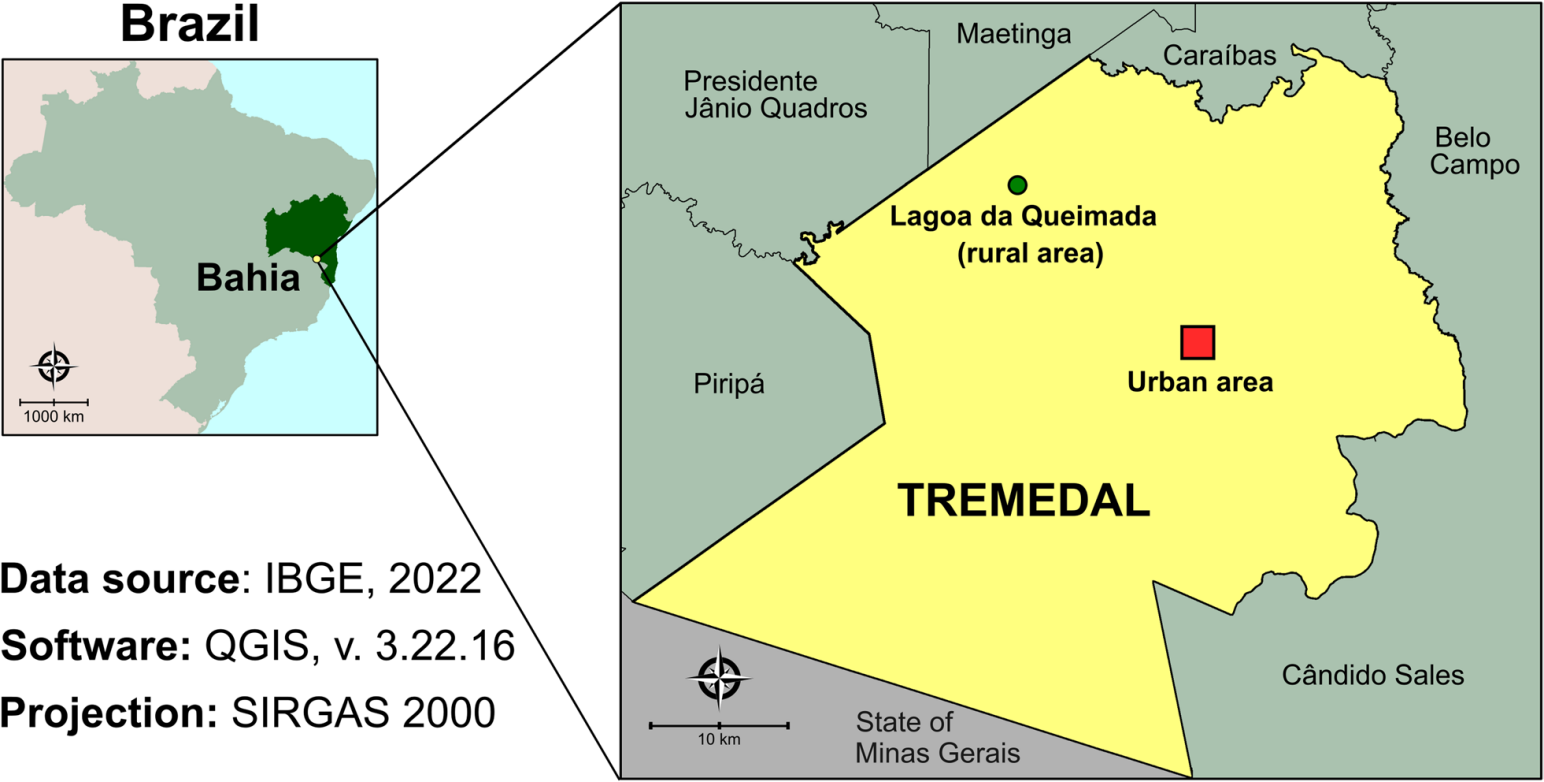
Map of Brazil showing the study area. A public domain digital map was obtained from the IBGE cartographic database in shapefile format (.shp), and then reformatted and analyzed using QGIS version 3.22.16 (Geographic Information System, Open-Source Geospatial Foundation Project: http://qgis.osgeo.org). IBGE: Brazilian Institute of Geography and Statistics

Serum samples were collected from domestic dogs as part of the municipal anti-visceral leishmaniasis canine campaign. Dogs of both genders that were aged ≥ 12 months and residing in urban or rural areas of Tremedal, Bahia were eligible for inclusion in the study. Samples were collected from 16 dogs near the Tremedal Supply Center (CEAT) and from one dog from Lagoa da Queimada, a rural area, due to a suspected case of leishmaniasis (Fig. 1). The dogs were selected by local agents engaged in endemic disease control and the municipal veterinarian. Although some dogs exhibited signs of malnutrition due to nomadic behavior, all were in stable clinical condition. Data on sex, estimated age (determined by the veterinarian), location and blood collection dates were recorded. Approximately 4 ml of blood was drawn from each dog via the cephalic vein between September and November 2022. The samples were centrifuged, and serum aliquots were stored at − 20 °C until serological tests were performed.

### *Leishmania* serology testing

The TR DPP® Canine Visceral Leishmaniasis Bio-Manguinhos test (Bio-Manguinhos/Fiocruz, Rio de Janeiro, RJ, Brazil) was used to detect anti-*Leishmania* spp. immunoglobulin G (IgG) antibodies. An aliquot of each sample was sent to the Central Laboratory of Public Health of Bahia (LACEN-BA) for confirmatory diagnosis using the enzyme-linked immunosorbent assay (ELISA) kit EIE-leishmaniose-visceral-canina-Bio-Manguinhos (EIE-LVC) (Bio-Manguinhos/Fiocruz, Rio de Janeiro, RJ, Brazil). Both tests were performed according to the manufacturers’ instructions. As per the National Program for Visceral Leishmaniasis Control, dogs were considered to be infected if the collected samples tested positive in both serological methods [18].

### Chagas disease serology testing

Anti-*T. cruzi* antibodies were detected using indirect ELISA with four *T. cruzi* chimeric recombinant antigens. These antigens were obtained following the methodology of Santos et al. [19].

The ELISA protocol was based on Leony et al. [20]. In detail, 96-well polystyrene microplates (Nunc Maxisorp®; Invitrogen, Thermo Fisher Scientific, Waltham, MA, USA) were coated with 100 μl of a carbonate/bicarbonate buffer (50 mM, pH 9.6) containing Instituto de Biologia Molecular do Paraná (IBMP) antigens IBMP-8.1, IBMP-8.2 and IBMP-8.4 at 25 ng per well, and IBMP-8.3 antigen at 50 ng per well. Stabilization was achieved using WellChampion™ solution (Ken-Em-Tec Diagnostics A/S, Taastrup, Denmark). After incubation at room temperature for 15 min, the microplates were dried at 37 °C for 3 h. Serum samples, diluted 1:100 in phosphate buffered saline (PBS), were added to each well (100 μl/well) and incubated at 37 °C for 1 h. Following incubation, the plates were washed with PBS-Tween 0.05% and incubated with anti-dog IgG conjugated with peroxidase (Bio-Manguinhos). After incubation and treatment with TMB Plus chromogen for color development, and the reaction was stopped with 0.3 M H_2_SO_4_. Optical density (OD) was measured at 450 nm on a SPECTRAmax 340PC® microplate reader (Molecular Devices, San Jose, CA, USA).

In the absence of a gold standard test for the diagnosis of CD, we applied a latent class analysis (LCA) model to classify the samples, following Fontes et al. [21]. Samples were classified as positive for CD if ≥ 2 chimeric antigens tested positive with a posteriori probability (PP) exceeding 68%. Samples testing positive for only the IBMP-8.1 and IBMP-8.3 antigens with PP < 50% were classified as negative for CD. Samples were also classified as negative if they exhibited no positive results or only one positive result with PP < 31%.

### Statistical analysis

Data analysis and visualization were performed using Prism software (version 9.5.1; GraphPad, San Diego, CA, USA). Descriptive statistics were presented as the median with interquartile ranges (IQR). Cutoff (CO) values for the IBMP-ELISA values were established by evaluating 10 *T. cruzi*-reactive and 10 *T. cruzi*-non-reactive samples [21]. The CO values were calculated using receiver operating characteristic (ROC) curves, and results were expressed as reactivity indices (RI). RI values > 1.00 were considered to be positive, while those within 1.0 ± 10% were classified as inconclusive. The prevalence of CD and LV was determined through serological testing. A digital map (Fig. 1) illustrating the study area was obtained from the Brazilian Institute of Geography and Statistics (IBGE) cartographic database and analyzed using QGIS version 3.22.16 (Geographic Information System, Open-Source Geospatial Foundation Project: http://qgis.osgeo.org).

## Results

A total of 17 dogs were tested for both anti-*Leishmania* spp. and anti-*T. cruzi* antibodies. The median age of the dogs was 60 months (IQR 30–72 months), with a female-to-male ratio of 1.4:1. As shown in Table 1, all sera samples were collected from dogs in urban areas, with the exception of dog #17, which was from the rural area of Lagoa da Queimada.

**Table 1:** Serological results of domestic dogs tested for *Trypanosoma cruzi* and *Leishmania infantum* in Tremedal, Bahia

Dog	Origin	Sex	Age (months)	Leishmaniasis			Chagas disease					
				DPP	EIA	Diagnostic result	IBMP antigen				LCA	
							8.1	8.2	8.3	8.4	Result	PP (%)
1	Urban	F	60	NEG	NEG	NEG	0.32	0.34	0.25	0.26	NEG	0.0
2	Urban	F	36	NEG	NEG	NEG	0.68	0.44	0.33	0.31	NEG	0.0
3	Urban	M	24	NEG	NEG	NEG	0.13	0.10	0.09	0.07	NEG	0.0
4	Urban	F	36	NEG	NEG	NEG	2.53	0.20	0.14	0.14	NEG	1.6
5	Urban	F	72	NEG	NEG	NEG	0.37	0.24	0.36	0.21	NEG	0.0
6	Urban	F	24	NEG	NEG	NEG	0.32	0.35	0.54	0.20	NEG	0.0
7	Urban	F	120	NEG	NEG	NEG	0.35	0.24	0.18	0.14	NEG	0.0
8	Urban	F	72	POS	NEG	NEG	0.31	0.23	0.18	0.13	NEG	0.0
9	Urban	F	72	POS	NEG	NEG	1.05	0.44	0.22	0.11	NEG	1.6
10	Urban	M	12	POS	NEG	NEG	2.51	0.37	0.23	0.22	NEG	1.6
11	Urban	M	60	NEG	NEG	NEG	0.26	0.26	0.26	0.17	NEG	0.0
12	Urban	M	120	NEG	NEG	NEG	0.36	0.32	0.09	0.12	NEG	0.0
13	Urban	M	36	POS	NEG	NEG	0.63	0.52	0.20	0.16	NEG	0.0
14	Urban	M	36	POS	NEG	NEG	0.28	0.08	0.10	0.06	NEG	0.0
15	Urban	F	24	POS	NEG	NEG	0.54	0.23	0.20	0.44	NEG	0.0
16	Urban	F	72	NEG	NEG	NEG	0.18	0.35	0.14	0.24	NEG	0.0
17	Rural	M	144	POS	POS	POS	2.77	1.22	0.39	0.61	POS	94.9

*DDP* TR DPP® Canine Visceral Leishmaniasis Bio-Manguinhos test, * EIA* EIE-leishmaniose-visceral-canina-Bio-Manguinhos ELISA Kit,* F* female, * IBMP* Instituto de Biologia Molecular do Paraná* LCA* latent class analysis,* M* male,* NEG* negative,* POS* positive, *PP* posteriori probability

*Leishmania* spp. exposure, detected using the TR DPP® Canine Visceral Leishmaniasis Bio-Manguinhos test, was found in 41.2% (7/17) of the dogs tested (Table 1). However, only one sample (5.9%; 1/17) tested positive using the confirmatory EIE Canine Visceral Leishmaniasis Bio-Manguinhos test, resulting in a seroprevalence of 5.9% (1/17). This positive sample came from a 12-year-old male dog residing in Lagoa da Queimada. The remaining six samples, which were DPP-positive only, were classified as false positive or inconclusive. The agreement between the TR DPP® and ELISA was poor, with a kappa (κ) value of 0.16.

All serum samples were tested for *T. cruzi* infection using LCA. Sixteen samples (94.1%) were classified as negative, while one sample (5.9%) was predicted to be positive for *T. cruzi* antibodies, indicating a CD prevalence of 5.9%. Table 1 presents the results for each IBMP antigen and the final LCA classification. The majority of samples (13/17; 76.5%) were negative across all four chimeric antigens. For three samples, the PP of being true positives was < 1.6%, confirming their classification as negative. The sample classified as positive by LCA showed reactivity to both IBMP-8.1 and IBMP-8.2 antigens, with a PP > 94.9%, suggesting a high likelihood of accurate classification. Notably, the only sample positive for *T. cruzi* antibodies also tested positive for *Leishmania* antibodies.

## Discussion

This study represents an initial effort to evaluate the seroprevalence of leishmaniasis and CD in dogs residing in the municipality of Tremedal, Bahia, as part of the Oxente Chagas Bahia Project [14]. Antibodies to *T. cruzi* and *Leishmania* spp. were detected in a single animal from a rural area, indicating a seroprevalence of 5.9% for both leishmaniasis and CD among the tested dogs. Despite the small sample size, these findings suggest that dogs in Tremedal are exposed to infected triatomine bugs and sandflies, confirming their role as natural sentinels and indicating the circulation of these parasites in the region.

Considering that Tremedal is classified as a high-risk area for human infection by *T. cruzi*, our findings emphasize the presence of *T. cruzi*-infected dogs, underscoring the importance of incorporating dogs into CD surveillance programs. The presence of infected dogs in domestic environments significantly elevates the risk of transmission to humans, potentially increasing the likelihood of *T. cruzi* infection compared to households with non-infected dogs [12].

To address the issue of serological cross-reactivity with other potentially co-endemic trypanosomatids, in the present study we used differential serological testing for *Leishmania* spp. on all dogs to avoid false positives. Using only the TR DPP® Canine Visceral Leishmaniasis Bio-Manguinhos test would have resulted in a sevenfold overestimation of leishmaniasis prevalence (41.2% samples tested positive). However, the confirmatory ELISA test revealed a much lower prevalence of 5.9%, highlighting the potential for false positives in the initial screening. The poor agreement between the DPP and ELISA test results (κ = 0.16) underscores the necessity for confirmatory testing to ensure accurate diagnosis and effective disease management. Conversely, our use of LCA provided a robust method for classifying samples in the absence of a gold standard test for CD. The classification criteria, based on PPs and multiple chimeric antigens, enabled accurate differentiation between reactive and non-reactive samples. This methodology serves as a valuable tool for application in future seroepidemiological studies in similar settings.

One animal tested positive for both *Leishmania* spp. and *T. cruzi*, underscoring the importance of controlling and monitoring both diseases in canine populations to reduce the risk of zoonotic transmission. Effective control measures for canine leishmaniasis involve vector control the use of topical repellents, treatment of infected dogs and education of pet owners on prevention practices. While there is no definitive cure for CD in dogs, current treatments aim to control clinical signs and prolong survival [22]. The veterinary community in endemic regions can play a critical role in vector control programs by encouraging the utilization of insecticides and elimination of vector habitats, conducting vector surveillance and promoting preventive practices among pet owners [23, 24].

The detection of *T. cruzi* antibodies in one dog of the 17 dogs tested indicates the ongoing presence of CD in the region, despite eradication efforts targeting *T. infestans*. The presence of other triatomine species capable of sustaining *T. cruzi* transmission highlights the need for continued vigilance and vector control measures. Due to the age of the one animal which tested positive, it is not possible to determine the mode of *T. cruzi* transmission—whether through insect ingestion, congenital transmission or vectorial transmission. Interestingly, the only dog testing positive for *T. cruzi* antibodies was also positive for anti-*Leishmania* antibodies, suggesting overlapping transmission cycles and shared environmental and vectorial factors contributing to the spread of both pathogens. These findings suggest that the detection of a single dog with a mixed infection, while no other dog tested positive for individual infections, may be influenced by factors such as the small sample size, differential vector exposure in rural areas, host susceptibility and prolonged environmental exposure, particularly in older animals with increased risk behaviors, underscoring the need for expanded surveillance to better characterize the epidemiology of *T. cruzi* and *L. infantum* co-infections in endemic regions. This co-infection warrants further investigation into the ecological and epidemiological dynamics of these infections in Tremedal.

Despite technological advancements in the diagnosis of CD and leishmaniasis in dogs [20, 21, 25, 26], the complexity of diagnosing and managing *Leishmania* spp. and *T. cruzi* infections in endemic areas is acknowledged and reinforced by our current findings. The high prevalence of false positives in initial screenings and the detection of co-infections emphasize the importance of using multiple diagnostic tools and confirmatory tests. Moreover, the continued presence of triatomine vectors in Tremedal calls for sustained surveillance and control efforts to mitigate the spread of these parasitic diseases. Future studies should focus on expanding the sample size and geographic coverage to gain a more comprehensive understanding of the epidemiological patterns of these pathogens. Additionally, exploring the genetic diversity of circulating parasite strains and their vectors could provide valuable insights into the factors driving transmission and persistence in the region.

## Conclusions

Overall, our study underscores the complexity of diagnosing and managing *Leishmania* spp. and *T. cruzi* infections in endemic areas. The high rate of false positives in initial screenings reinforces the need for confirmatory tests and the use of multiple diagnostic tools. Moreover, the persistent presence of triatomine vectors in Tremedal necessitates ongoing surveillance and control efforts. Future research should expand sample size and geographic scope to further understand the epidemiology of *Leishmania* spp. and *T. cruzi* infections. Including regions such as Novo Horizonte, Bahia, a medium-risk area for *T. cruzi* infection and part of the Oxente Chagas Bahia Project, in such studies will provide valuable insights and contribute to more effective disease control strategies.

## Data Availability

No datasets were generated or analysed during the current study.

## Abbreviations

CD: Chagas disease
CEAT: Tremedal Supply Center
CO: Cutoff
ELISA: Enzyme-linked immunosorbent assay
IBMP: Instituto de Biologia Molecular do Paraná
LACEN-BA: Central Laboratory of Public Health of Bahia
LCA: Latent class analysis
PBS: Phosphate buffered saline
PP: Posteriori probability
RI: Reactivity index
VL: Visceral leishmaniasis
